# Medullary carcinoma of the pancreas radiologically followed up as a cystic lesion for 9 years: a case report and review of the literature

**DOI:** 10.1186/s40792-018-0487-3

**Published:** 2018-07-24

**Authors:** Akikazu Yago, Mitsuko Furuya, Ryutaro Mori, Yasuhiro Yabushita, Yu Sawada, Takafumi Kumamoto, Ryusei Matsuyama, Michio Shimizu, Itaru Endo

**Affiliations:** 1 Department of Gastroenterological Surgery, Yokohama City University Graduate School of Medicine, Yokohama, Japan; 2 Department of Molecular Pathology, Yokohama City University Graduate School of Medicine, 3-9 Fukuura, Kanazawa-ku, Yokohama, 236-0004 Japan; 3 Diagnostic Pathology Center, Hakujikai Memorial Hospital, Tokyo, Japan

**Keywords:** Medullary carcinoma of the pancreas, *KRAS* mutation, Microsatellite instability

## Abstract

**Background:**

Medullary carcinoma of the pancreas (MCP) is a rare malignant pancreatic tumor. The World Health Organization classification defines the tumor as a subtype of pancreatic ductal carcinomas. MCP is histologically characterized as having highly pleomorphic cells with syncytial morphology, expansive tumor growth, and necrosis. The pathogenesis and clinical course of MCP are largely unknown. Herein, we report an unusual case of MCP that was radiologically followed up for 9 years prior to surgical intervention.

**Case presentation:**

A 73-year-old Japanese woman with a nonspecific disease history was found to have an asymptomatic cyst in the pancreatic duct by abdominal ultrasonography. Thorough radiological investigation suggested it was not an aggressive neoplasm, and she received periodic check-ups under a clinical diagnosis of “a cystic pancreatic lesion of uncertain malignancy.” Nine years after initial presentation, she experienced acute pancreatitis and underwent thorough re-evaluation. Dynamic computed tomography revealed no cyst; rather, a solid tumor was detected. Cytology of the pancreatic duct suggested adenocarcinoma. Pancreatoduodenectomy with D2 lymph node dissection was performed. The resected tumor was a non-mucinous, solid mass measuring 22 × 10 mm. Microscopically, the tumor had a well-demarcated pushing border. Lymphocytic infiltration was abundant, and stromal component was sparse. The tumor cells were composed of highly pleomorphic cells, proliferating in sheets without glandular formation. Neither lymphovascular invasion nor lymph node metastasis was detected. The histopathologic diagnosis was MCP, pT1aN0M0. The tumor carried a *KRAS* mutation, and MLH-1, MSH-2, MSH-6, and PMS-2 immunostaining results were normal, suggesting microsatellite stability. The patient has remained free of disease for 29 months following surgical intervention.

**Conclusion:**

A review of 20 previously reported cases plus the present case suggests that subsets of MCPs have genetic aberrations such as *KRAS* mutation and high microsatellite instability. MCP has been suggested to have a better prognosis than common ductal adenocarcinoma; however, 15 of 20 previously reported cases died from disease. Whether the asymptomatic cyst observed over 9 years contributed to the development of MCP in this patient is a subject for future study.

**Electronic supplementary material:**

The online version of this article (10.1186/s40792-018-0487-3) contains supplementary material, which is available to authorized users.

## Background

Medullary carcinoma of the pancreas (MCP) is a rare type of pancreatic malignancy. It is defined as a subtype of pancreatic ductal carcinoma according to the World Health Organization (WHO) classification. MCP is histologically characterized as a poorly differentiated tumor with extensive necrosis, a syncytial growth pattern, and a pushing border [1]. Only a few papers have described the pathologic features of MCP, in which the tumor occasionally develops in the background of Lynch syndrome [2]. The prognosis of patients with MCP has been suggested to be better than that of patients with more common ductal adenocarcinomas; however, very little is known about the pathogenesis and molecular mechanisms of this disease. Herein, we present an unusual case of MCP that was surgically resected after 9 years of watchful waiting with a radiologic diagnosis of “a cystic lesion of uncertain malignancy.” We describe radiologic, surgical, and pathologic findings of this case. We also review previously reported 20 previously reported MCP cases and discuss the clinicopathologic features of this rare malignancy.

## Case presentation

A 73-year-old Japanese female with a history of diabetes mellitus, hypertension, and hyperlipidemia was found to have a cystic lesion in the pancreas by abdominal ultrasonography. Her mother had died of gastric cancer, and her aunt had died of pancreatic cancer. Although she previously had a benign colon polyp, the family history and medical records did not meet the Amsterdam criteria II for Lynch syndrome. The asymptomatic cyst of the pancreas was periodically checked. Over 7 years, the cyst slowly enlarged and was radiologically suspected to be an intraductal papillary mucinous neoplasm (IPMN). At 9 years, she presented with a dull feeling in the stomach and was diagnosed with acute pancreatitis. She received medical treatment then was referred to our clinic for further examination.

**Fig. 1:**
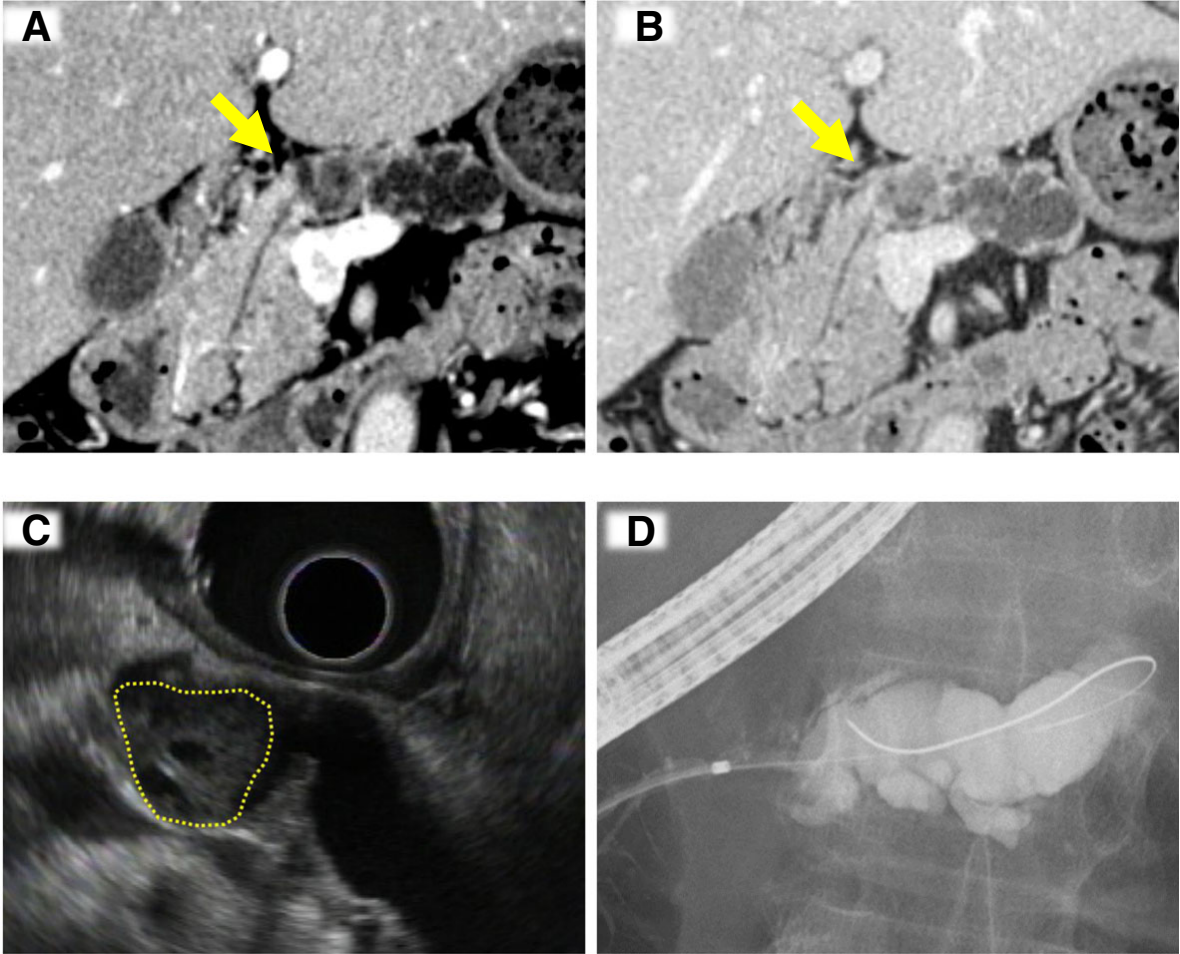
Radiologic findings. **a**, **b** Dynamic computed tomography. A solid mass in the pancreatic body (indicated by an arrow) is enhanced at the arterial phase (**a**), and the enhancement is prolonged at the delayed phase (**b**). **c** Endoscopic ultrasonography. A tumor developed in the main pancreatic duct (a dotted circle). **d** Endoscopic retrograde cholangiopancreatography. The main pancreatic duct of the distal portion is markedly dilated because the solid tumor obstructs fluid passage

Dynamic computed tomography and endoscopic ultrasonography revealed no cyst. Instead, a solid tumor was observed in the main pancreatic duct of the pancreatic body (Fig. 1a–d). The main duct of the pancreatic tail was dilated due to obstruction. The tumor was enhanced from the early to delayed phases. Cytology from the pancreatic duct by endoscopic retrograde cholangiopancreatography indicated an adenocarcinoma; however, mucous secretion was not detected, suggesting that IPMN was unlikely. The maximum standardized uptake value of the lesion was 6.8 by positron emission tomography (Additional file 1). On laboratory examination, hematologic and biochemical data values were all within normal ranges. Serum levels of carcinoembryonic antigen (CEA), carbohydrate antigen (CA) 19-9, and pancreatic cancer-associated antigens (DUPAN-2 and SPAN-1) were all within normal ranges.

The pre-operative diagnosis was invasive ductal adenocarcinoma, and the patient underwent pancreatoduodenectomy and D2 lymph node dissection. The clinicopathologic diagnosis was stage I ductal adenocarcinoma. The surgical margin was free of tumor cells. No lymph node or distant metastasis was detected; thus, the tumors-nodes-metastasis (TNM) stage was pT1aN0M0. The postoperative course was uneventful, and the patient was discharged at postoperative day 22 without comorbidity. The patient received six courses of S-1 (100 mg/day) as the postoperative adjuvant therapy. She has undergone medical check-ups every month including tumor markers (CEA and CA19-9) bimonthly. Computed tomography and ultrasonography have been done every 3 months. Although CEA has fluctuated between 4 and 11 ng/ml, no evidence of recurrence has been detected so far.

**Fig. 2:**
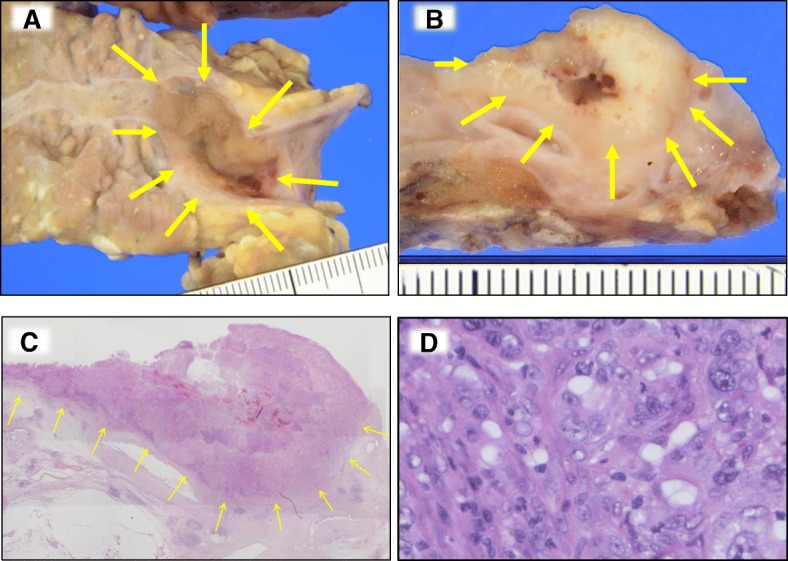
Pathologic findings. **a**–**c** The resected tumor (indicated by arrows). A solid mass developed in the pancreatic duct (**a**). Cut surface of the pancreatic duct demonstrates a well-demarcated whitish mass (**b**). The tumor has a pushing border in the loupe image (**c**). **d** Medullary growth with prominent lymphocytic infiltration is observed. The tumor contains some syncytial cells

The resected specimen contained a 22 × 10-mm circumscribed nodular tumor in the pancreatic main duct (Fig. 2a–d). Microscopically, the tumor grew in an expansive manner. The border between the tumor and non-tumor areas was well defined and associated with prominent lymphocytic infiltration. Contrary to typical invasive ductal adenocarcinomas characterized by tubular structures with abundant stroma, the tumor cells proliferated in a medullary pattern, with fewer stromal components. Close examination revealed marked nuclear pleomorphism with prominent nucleoli. Syncytial cells were observed sparsely. Although the pancreas had been radiologically followed for 9 years due to the presence of a suspected cystic lesion, the pancreas did not actually have any cysts. The distal portion of the pancreatic duct was dilated due to obstruction, but no neoplastic/metaplastic changes were observed. Immunohistochemical analysis revealed that the tumor was positive for cytokeratin (CK)-7 and CK-20, and focally positive for mucin (MUC) 5AC and MUC6. The tumor was negative for MUC2 and caudal-type homeobox (CDX) 2 (Additional file 2). These staining patterns supported neither IPMN nor intraductal tubulopapillary neoplasm. Based collectively on these observations, the tumor was diagnosed as MCP.

Possible contribution of oncogenic gene mutations and microsatellite instability (MSI) to MCP carcinogenesis is mentioned in the literature; therefore, further pathologic characterization was performed. A *KRAS* codon 12 mutation (G12V) was detected in the tumor tissue. Immunohistochemical analysis for MutL homolog (MLH) 1, MutS homolog (MSH) 2, MSH6, and postmeiotic segregation increased (PMS) 2 demonstrated normal positive staining patterns, indicating microsatellite stability. Together with the family history that did not match the Amsterdam II/Bethesda criteria, Lynch syndrome was clinically ruled out. In situ hybridization for Epstein-Barr virus (EBV) peptide nucleic acid was negative.

Only a few papers have described the histopathologic characteristics and clinical outcomes of MCP, and the pathogenesis of this malignancy is largely unknown. Clinicopathologic features of 20 MCPs in the literature and the present case are summarized in Table 1 [1–4]. Although a better prognosis compared to usual ductal adenocarcinoma of the pancreas is suggested, 15 patients died of disease (70%), 11 within 1 year of diagnosis. This indicates that MCP essentially has a poor prognosis, even though some patients experience longer survival compared to patients with more common ductal adenocarcinomas. Six patients survived for ≥ 24 months, and two survived for ≥ 5 years. The present patient has been followed for 29 months without recurrence, which may be due in part because she underwent surgical intervention of stage I disease, after watchful waiting for 9 years. None of previous MCPs was reported to have watchful surveillance prior to surgery.

**Table 1:** Literature review of clinical and genetic characteristics of 21 medullary carcinomas of the pancreas

Case	Authors (reference no.)	Age	Sex	*MSI	Family history	*KRAS* mutation	Status (months)
1	Goggins et al. [1]	71	M	+	+	Wild	Alive (67)
2		84	F	+	+	Wild	DOD (4)
3		72	M	+	+	Wild	Alive (24)
4		85	M	−	+	G12D	DOD (40)
5		65	M	−	+	G12R	DOD (9)
6	Wilentz et al. [2]	72	M	−	+	G12R	DOD (0)
7		74	F	−	Unknown	Wild	DOD (8)
8		79	M	−	+	G12D	DOD (5)
9		53	F	−	+	Wild	DOD (45)
10		44	M	−	Unknown	Wild	DOD (11)
11		49	M	−	Unknown	Wild	DOD (15)
12		74	M	−	+	Wild	DOD (12)
13		74	F	−	Unknown	Wild	DOD (12)
14		34	M	+	+	Wild	Alive (13)
15		33	M	−	−	Wild	Alive (126)
16		67	F	−	+	G12F	DOD (15)
17		67	M	−	+	G12D	DOD (4)
18		66	F	−	+	Wild	DOD (7)
19	Banville et al. [3]	63	M	+	+	Not done	Unknown
20	Cumplido Buron and Toral Pena [4]	59	M	Not done	Unknown	Wild	DOD (5)
21	The present case	73	F	− (IHC)	−	G12 V	Alive (29)

*MSI* microsatellite instability, *DOD* died of the disease, *IHC* immunohistochemistry; *MSI in cases 1–19 was examined by IHC and microsatellite markers/Sanger sequencing. In case 21, MSI was examined by IHC only

Previous studies of MCP suggest two possible genetic pathways: *KRAS* mutation and MSI. In the literature, four of five high-MSI cases had wild-type *KRAS*, whereas all *KRAS*-mutant MCPs (*n* = 7) showed microsatellite stability (Table 1). These findings indicate that aberrations in these two pathways may occur in a mutually exclusive manner in patients with MCPs. According to The Cancer Genome Atlas (TCGA) network analyses of typical pancreatic ductal carcinomas, *KRAS* mutation is the most frequent genomic event (140/150, 93%) while MSI is not observed [5]. Therefore, MSI in the five MCP patients should be noted as a potentially specific event. In contrast, *KRAS* mutation alone cannot distinguish MCP from typical pancreatic ductal adenocarcinomas. Four of seven patients with *KRAS*-mutant MCP died within 1 year; these outcomes are similar to those of patients with advanced typical ductal adenocarcinomas. If *KRAS*-mutant MCPs share other common molecular signatures with typical ductal adenocarcinomas, such as *TP53* mutation and copy number losses of cyclin-dependent kinase inhibitor (*CDKN*) *2A* and *SMAD4* [5, 6], characteristic histology in these cases may depend on inflammatory events unrelated to genetic background. One case of microsatellite-stable MCP showed positivity for EBV RNA [2]. The current patient experienced an episode of acute pancreatitis. In situ hybridization for EBV was negative, suggesting that EBV infection was not associated with this case. Although the WHO classification of MCP is based on morphological characteristics such as pushing growth of highly pleomorphic carcinoma cells, including syncytial cells, no specific markers have been identified that distinguish *KRAS*-mutant MCP from undifferentiated/poorly differentiated ductal adenocarcinomas. Together with genomic divergence, inflammation-related episodes in a few MCPs allow us to hypothesize that MCP potentially consists of more than one subtype and that adventitious events such as acute infection may contribute to the histopathology. Detailed molecular characterization using larger numbers of MCP cases should be conducted in future studies.

## Conclusions

We have presented a case of MCP that developed from an asymptomatic cystic lesion present for 9 years and was resected after an episode of acute pancreatitis. The tumor carried a *KRAS* mutation and lacked MSI. A literature review of 20 MCPs plus the present case revealed two distinctive genomic alteration types that seem to occur in a mutually exclusive manner, i.e., *KRAS* mutation and MSI. Most patients have poor prognoses. We hope that our study will contribute to a better understanding of the pathogenesis of this rare variant of pancreatic ductal adenocarcinoma.

## Additional files

Additional file 1: Positron emission tomography (PET). The maximum standardized uptake value of the preoperative lesion was 6.8 (arrows). (PPTX 120 kb)

Additional file 2: Immunohistochemical staining. The tumor was positive for cytokeratin (CK)-7 and CK-20, and focally positive for mucin (MUC) 5 AC and MUC6. The tumor was negative for MUC2 and caudal-type homeobox (CDX) 2. (PPTX 9523 kb)

## Abbreviations

CA: Carbohydrate antigen
*CDKN*: Cyclin-dependent kinase inhibitor
CDX: Caudal-type homeobox
CEA: Carcinoembryonic antigen
CK: Cytokeratin
DOD: Died of disease
EBV: Epstein-Barr virus
IHC: Immunohistochemistry
IPMN: Intraductal papillary mucinous neoplasm
MCP: Medullary carcinoma of the pancreas
MLH: MutL homolog
MSH: MutS homolog
MSI: Microsatellite instability
MUC: Mucin
PMS: Postmeiotic segregation increased
TCGA: The Cancer Genome Atlas
TNM: Tumors-nodes-metastasis
WHO: World Health Organization
