# Supplementary figures and images for: Medullary carcinoma of the pancreas radiologically followed up as a cystic lesion for 9 years: a case report and review of the literature
Source: Surg Case Rep. 2018 Jul 24;4:80. doi: 10.1186/s40792-018-0487-3 (PMC6057860; doi:10.1186/s40792-018-0487-3)

## Slide 1
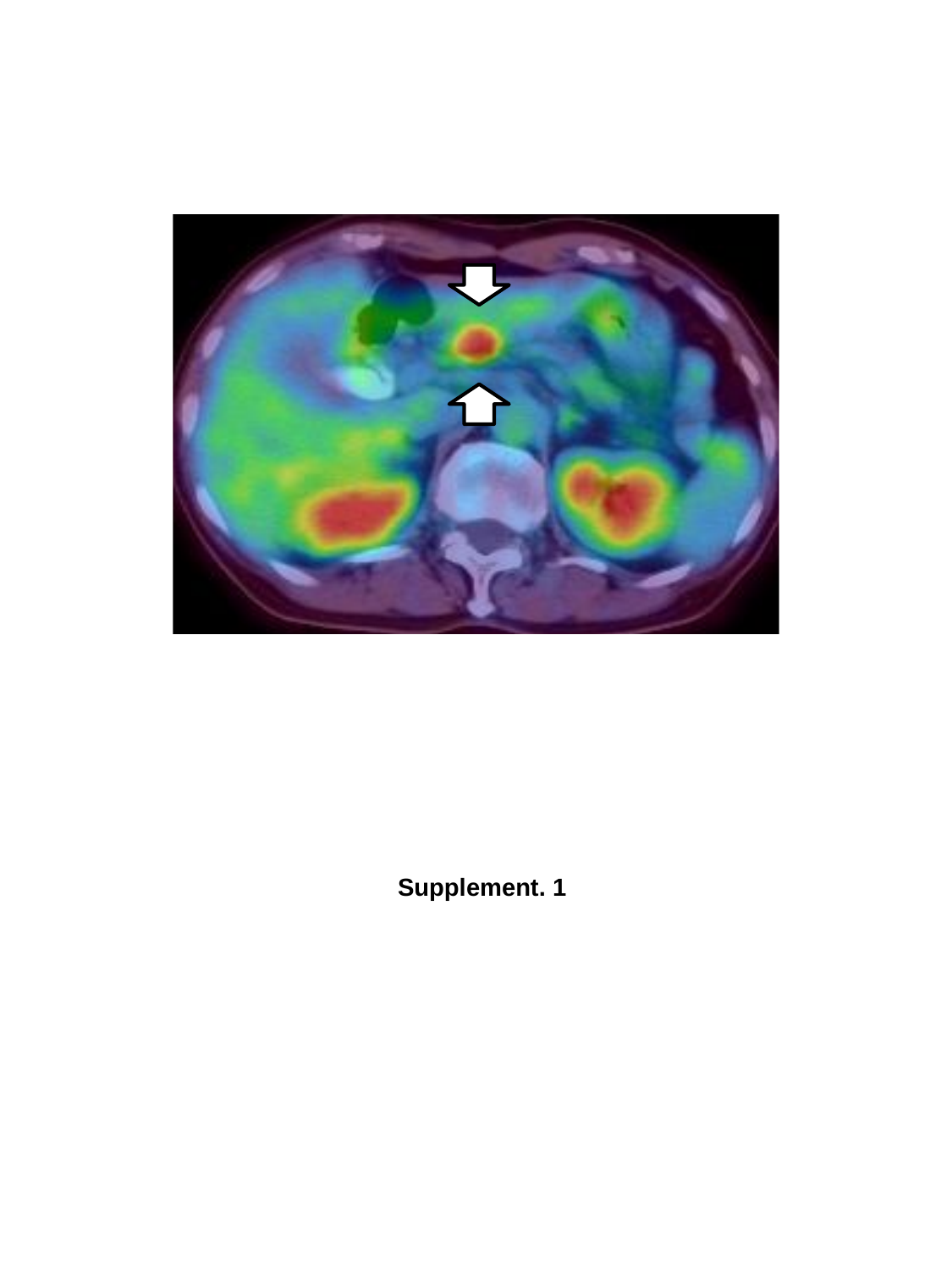

Supplement. 1

Supplement: Supplementary file 1 — Positron emission tomography (PET). The maximum standardized uptake value of the preoperative lesion was 6.8 (arrows). (PPTX 120 kb) [file 40792_2018_487_MOESM1_ESM.pptx]

## Slide 1
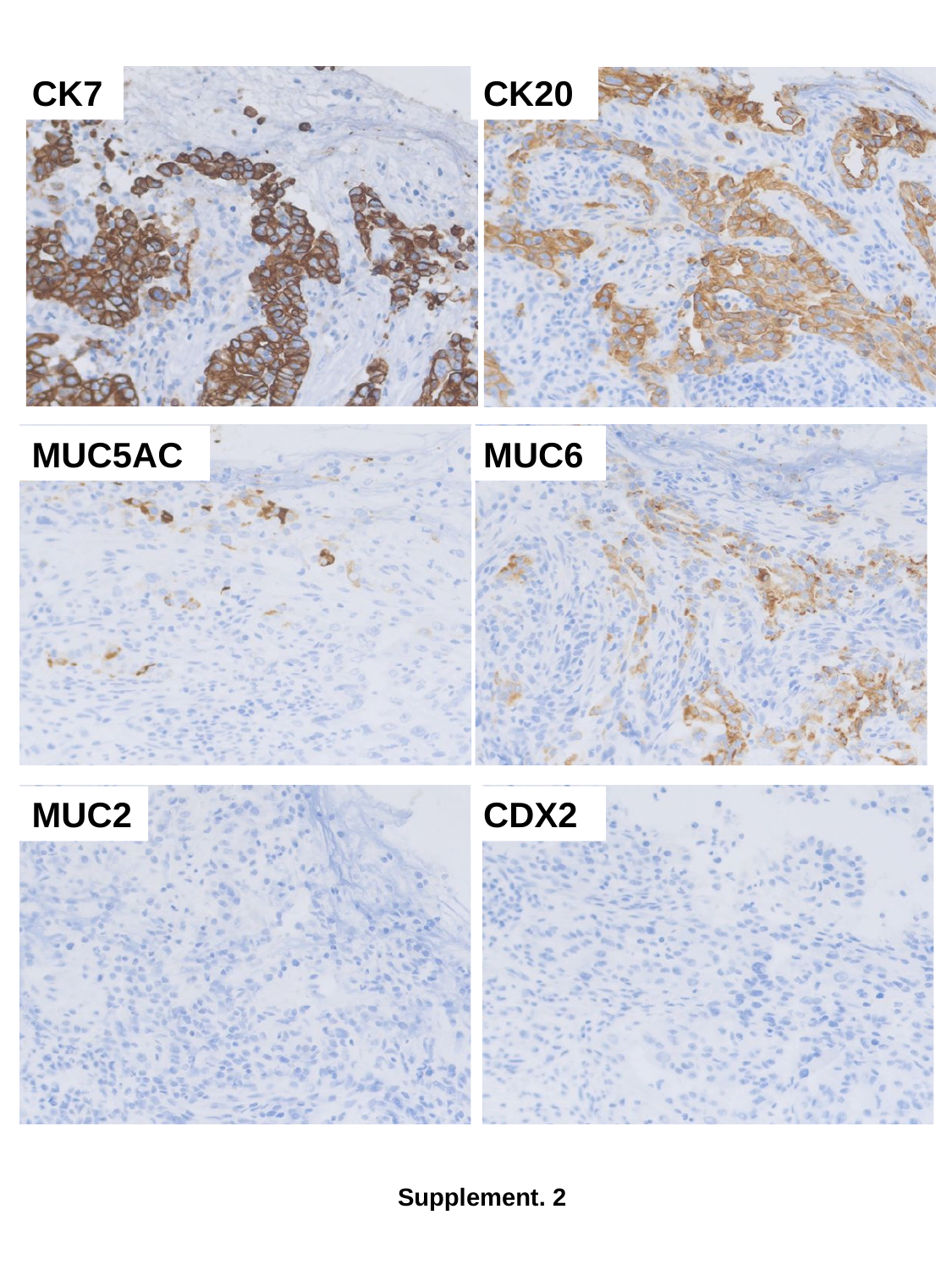

CK7
CK20
MUC5AC
MUC6
MUC2
CDX2
Supplement. 2

Supplement: Supplementary file 2 — Immunohistochemical staining. The tumor was positive for cytokeratin (CK)-7 and CK-20, and focally positive for mucin (MUC) 5 AC and MUC6. The tumor was negative for MUC2 and caudal-type homeobox (CDX) 2. (PPTX 9523 kb) [file 40792_2018_487_MOESM2_ESM.pptx]
